# The Flare of Rheumatic Disease After SARS-CoV-2 Vaccination: A Review

**DOI:** 10.3389/fimmu.2022.919979

**Published:** 2022-07-04

**Authors:** Yan Xie, Yang Liu, Yi Liu

**Affiliations:** ^1^ Department of Rheumatology and Immunology, West China Hospital, Sichuan University, Chengdu, China; ^2^ Tsinghua Clinical Research Institute (TCRI), School of Medicine, Tsinghua University, Beijing, China

**Keywords:** COVID-19, SARS-CoV-2, vaccine, rheumatic disease, flare

## Abstract

As the coronavirus disease 2019 (COVID-19) pandemic continues worldwide, vaccination has been considered an effective measure to protect people from the COVID-19 and end the pandemic. However, for patients with rheumatic diseases (RD), concern for the induction of RD flare may combat the enthusiasm for vaccination. In general, current evidence doesn’t support the increased risk of disease flare after COVID-19 vaccination. However, the disease flare of RDs may be triggered by COVID-19 vaccinations, especially for patients with high disease activity. Most of these flares after vaccination are mild and need no treatment escalation. Considering the benefits and risks, RD patients are recommended to receive the COVID-19 vaccination but should be vaccinated when the RDs are in stable states.

The coronavirus disease 2019 (COVID-19), caused by infection of the severe acute respiratory syndrome coronavirus 2 (SARS-CoV-2), has generated more than five million deaths worldwide (1). As there are still more than a million new cases confirmed daily, the COVID-19 pandemic remains a global threat to humanity (1). To end the pandemic, vaccination has been considered an effective measure. For patients with rheumatic diseases (RD), the American College of Rheumatology (ACR) recommended a priority of receiving vaccination based on the possibility of increased infection risk and severe outcomes of COVID-19 (2, 3). However, a recently published high-quality meta-analysis revealed that patients with RD do not face more risk of contracting SARS-CoV-2 or worse prognosis of COVID-19, which may partly combat the enthusiasm for vaccination in these patients (4).

Besides, many patients may also refuse or hesitate to be vaccinated mainly due to safety concerns, especially the risk of RD flare or relapse after vaccination (5). Thus, understanding the association between disease flare of RD and vaccination is essential to overcome vaccine hesitancy and increase the protection rate.

Theoretically, the risk of disease flare or worsening for RD patients does exist after COVID-19 vaccination. Infectious agents are always considered environmental triggers of autoimmunity for autoimmune diseases (6). The SARS-CoV-2 infection also shares similar molecular networks with RDs and triggers cross-reactivity through molecular mimicry, leading to autoimmunity (7). Vaccines, which contain antigens from these infectious agents, may also induce autoimmunity by similar mechanisms such as molecular mimicry, epitope spreading, bystander activation, and polyclonal activation (8). Except for antigens, adjuvants in the vaccine can also induce autoimmunity through various mechanisms (8, 9). To date, three types of vaccines, including messenger RNA (mRNA) vaccine, adenovirus-based vaccine, and inactivated vaccine, are generally available in different countries (10). The mRNA can serve as both immunogen and adjuvants for mRNA vaccines, stimulating innate immunity by activating the endosomal and cytosolic pattern-recognition receptors (PRRs) (11). For an adenovirus-based vaccine, the DNA contained in the virus particle can also stimulate the PRRs (11). The activated PRRs, such as the toll-like and RIG-I-like receptors, can subsequently trigger the intracellular signaling cascades, leading to inflammasome activation and type I interferon production (12). The implication of the type I interferon pathway has been shown in many rheumatic diseases, such as systemic lupus erythematosus (SLE), systemic sclerosis (SSc), and rheumatic arthritis (RA) (13). Therefore, the interactions between the immune responses to vaccination and RDs raise the concern that vaccination may induce disease flares. A study conducted by Ntouros et al. found that the mRNA vaccine against SARS-CoV-2 can lead to a transient increase of DNA damage *via* oxidative stress. Compared with healthy volunteers, significantly increased DNA damage formation and impaired repairing capacity were observed in SLE patients (14). The augmented DNA damage accumulation can induce autoantibody production and type I interferon-induced immune activation, which may finally facilitate the progression of systemic autoimmune diseases (15).

## Flare Rate

**Figure 1:**
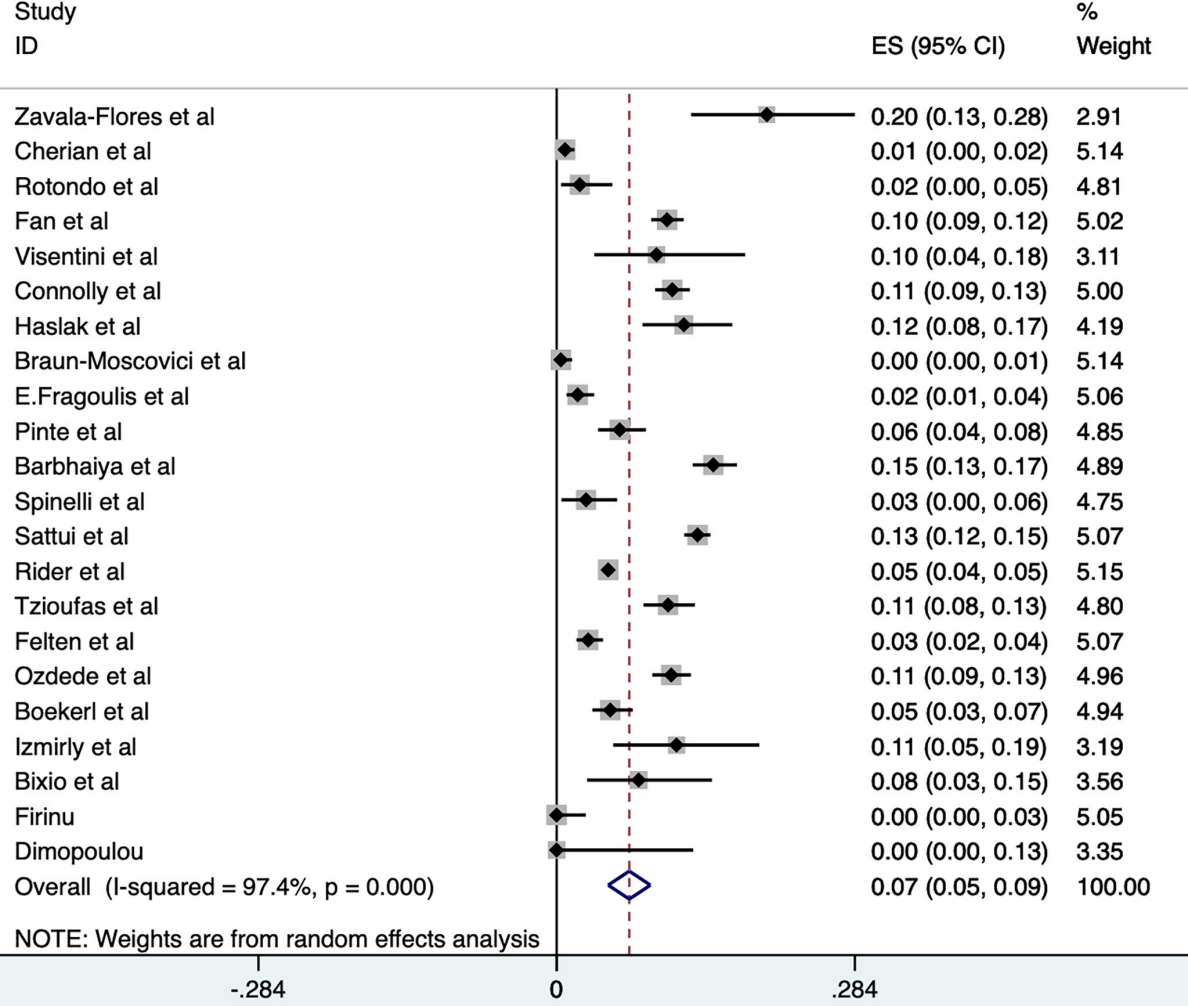
Total flare rate of rheumatic disease after COVID-19 vaccination.

Some observational studies have reported the flare rate of RDs after COVID-19 vaccination, ranging from 0.4% to 20% (16–37) (**Table 1**). A meta-analysis of these studies revealed that the overall random-effects rate of flare after COVID-19 vaccination was 7% (95%Cl, 5%-9%; *P*=0.000) in RDs patients. Not surprisingly, a high level of heterogeneity existed for this result (*P*=0.000, *I^2 ^= *97.4%) (**Figure 1**).

**Table 1:** Summary of studies on a flare-up of RDs after COVID-19 vaccination.

Author	Diseases	Vaccine Type	Flare Rate, % (n/N)			Common Flare Symptoms	Risk Factors	Protective Factors	Medication Change	Rate or Hospitalization for Flare
			Total	After 1^st^ dose	After 2^nd^ dose					
Zavala-Flores	SLE	mRNA	20% (20/100)	9% (9/100)	20% (18/90)	arthritis, dermal, leukopenia, myopericarditis, lupus pneumonitis	history of renal involvement, HCQ, AZA, flare history within 6 months	/	/	10% (2/20)
Cherian	RA (43.85%); SLE (10.13%); SpA (13.25%); Vasculitis (6.23%); inflammatory polyarthritis (15.59%); other (10.89%)	adenovirus-based, inactivated	0.8% (4/523)	/	/	arthritis	/	/	/	/
Rotondo	arthritis (78%); CTD (18%); vasculitis (4%)	adenovirus-based, mRNA	2.2% (3/137); mRNA:3/107 adenovirus-based:0/30	2.2% (3/137)	0	/	/	/	/	/
Fan	SLE (40.7%); RA (28.8%); BD (8.1%); PsA (5%);pSS (4.9%); AS (2.9%)	inactivated	10.5% (158/1507)	/	/	arthritis, skin rash, fever	elderly, allergic history	stable disease	3.5% (53/1507)	/
Visentini	Vasculitis	mRNA	9.5% (6/63)			Purpura, peripheral neuropathy				
Connolly	arthritis (47%); SLE (20%); SS (5%); vasculitis (3%); SSc (1%); overlap CTD (20%)	mRNA	11% (151/1377)	4.4% (61/1377)	7% (90/1377)	arthritis, muscle pain and weakness	prior infection, flare in the past 6 months, use of combination therapy	cDMARDs, biologics	2.5% (35/1377)	0 (0/151)
Haslak	JIA (24.2%); FMF (56.5%); CTD (13.5%); Vasculitis (4); other (1.8%)	mRNA, inactivated	12.1% (27/223)	10.3% (23/223) mRNA:20/198 inactive:3/28	3.1% (7/223) mRNA:7/198	arthritis or arthralgia, fever, cutaneous involvement	/	/	/	/
Braun-Moscovici	inflammatory arthritis (58%); CTD (34%); vasculitis (7%); other (2%)	mRNA	0.4% (1/264)	/	/	arthritis	/	/	/	/
E.Fragoulis	inflammatory arthritis (58.1%); CTD (27.5%); vasculitis (10.5%); other (3.9%)	adenovirus-based, mRNA	2% (9/441)	0.23% (1/441)	1.81% (8/441)	/	discontinuation of treatment	/	/	/
Pinte	RA (15.7%); SLE (15.6%); SS (12.5%); AS (10.4%); PsA (8.5%); scleroderma (5.0%); other (9.1%)	adenovirus-based, mRNA	6% (25/416) mRNA: 21/371 adenovirus-based:4/37	0.7% (3/416)	5.3% (22/416)	/	more than one immune disease, corticosteroids, history of flare-up during the previous year	/	5% (21/416)	40% (10/25)
Barbhaiya	SRDs	adenovirus-based, mRNA	14.9% (165/1101)	10.6% (117/1101) mRNA:114/1080 adenovirus-based:3/19	13.6% (85/626) all in mRNA	arthritis or arthralgia, myagia, fatigue, skin rash	/	/	/	/
Spinelli	RA (24.6%); SLE (24.6%); PsA (20.6%); UCTD (8.7%); AS (7.1%); other (14.3%)	mRNA	2.8% (3/126)	/	/	arthritis	/	/	/	/
Sattui	RA (42.3%); myositis (17%); SS (15.3%); SLE (13.7%); SpA (16.2%); vasculitis (5.8%); scleroderma (4.4%) other (4.2%)	adenovirus-based, mRNA	13.4% (382/2860) mRNA:280/2132 adenovirus-based:100/695	/	/	/	/	/	4.6% (132/2860)	/
Rider	RA (30.3%); IIMs (14.7%); SLE (14.1%); SS (9.6%); PsA (5.4%); AS (5.2%); other (8.9%)	adenovirus-based, mRNA, other	4.9% (274/5619) mRNA:190/4063 adenovirus-based:76/1200 other:8/356	/	/	/	Oxford AstraZeneca vaccine, female, SLE, PsA, PMR, prior serious reaction to non-COVID-19 vaccine	IMs	/	/
Tzioufas	RA (27.8%); SLE (19.5%); seronegative arthritis (20.8%); vasculitis (11.1%); SS (9.6%); IIMs (4.6%); other (11.4%)	mRNA	10.6%(64/605)	/	/	/	/	/	/	/
Felten	SLE	adenovirus-based, mRNA, inactivated	3.0% (21/696)	/	/	fever, cutaneous flare, musculoskeletal, fatigue	flare history during the past year	/	2.1% (15/696)	19% (4/21)
Ozdede	BD (23.6%); FMF (22.4%); RA (13.4%); SLE (8.0%); AS (14.5%); vasculitis (7.7%) other (10.7%)	mRNA, inactivated	10.9% (120/1104) mRNA:68/562 inactivated: 52/542	/	/	skin-mucosal lesion, joint symptoms	BD, FMF, experience any AE	/	/	/
Boekel	RA (40.4%) MS (16.0%)	mRNA, adenovirus-based	5.1% (26/505) mRNA:12/274 adenovirus-based:14/231	/	/	/	/	/	/	/
Izmirly	SLE	mRNA, adenovirus-based	11.4% (9/79)	1	8	thrombocytopenia, arthritis	/	/	/	/
Bixio	RA	mRNA	7.8% (6/77)	1	5	/	/	/	0	0
Firinu	RDs	mRNA	0 (0/102)	/	/	/	/	/	/	/
Dimopoulou	JIA	mRNA	0 (0/21)	/	/	/	/	/	/	/

AE, adverse events; AS, ankylosing spondylitis;AZA, azathioprine; BD, Becet’s disease; csDMARDs, conventional disease-modifying antirheumatic drugs; FMF, familial mediterranean fever; HCQ, hydroxychloroquine; IIMs, idiopathic inflammatory myositis; JIA, juvenile idiopathic arthritis; mRNA, messenger RNA; MS, multiple sclerosis; PMR, polymyagia rheumatica; PsA, psoriatic arthritis; pSS, primary sjogren’s syndrome; RA, rheumatic arthritis; RD, rheumatic disease; SLE, systemic lupus erythematosus; SpA, spondyloarthropathy; UCTD, undifferentiated connective tissue disease.

## Flare Rate in Different Vaccine Types, Doses, and Diseases

### Vaccine Type

As the type of vaccine used in different studies varied, a question of whether the heterogeneity came from the difference in vaccine types was raised. Results of our meta-analysis revealed similar flare rates of RDs after mRNA vaccination and after adenovirus-based vaccination, which were 7% (95%Cl, 5%-9%; *P*=0.000) and 8% (95%Cl, 4%-12%; *P*=0.000), respectively (**Supplementary Materials**). Pinte et al. made direct comparisons between several different mRNA and adenovirus-based vaccines, finding no difference in terms of flare-up development (*p*=0.43) (25). Only 37 patients in this study received adenovirus-based vaccination, while the number of patients receiving mRNA vaccination was 371; the huge distinction in sample sizes raises the concern of unreliability. Another study conducted by Sattui et al. involved 2132 RD patients who received mRNA vaccination and 695 RD patients who received adenovirus-based vaccination. In this study, the flare rate was also similar between the two groups (28). On the other hand, a multivariable logistic regression analysis conducted by Rider et al. found that adenovirus-based vaccine was associated with higher flare risk relative to mRNA vaccine (OR1.44, 95% Cl 1.08-2.48) (29). The results of Ozdede et al. also supported this (32). As for comparing mRNA and inactive vaccines, neither showed any prone to disease flare, as similar flare rates between the two groups were observed (22, 32). Notably, most studies involved are about mRNA vaccine, and data relevant to the flare rate of RDs after inactive and adenovirus-based vaccination are still minimal.

### RD Type

Different RDs can have different risks of disease flare. SLE, the typical representative of RDs, showed a flare rate ranging from 3% to 20% (16, 19, 21, 29, 31). Patients with RA, another common RD, shared similar flare rates in several studies (11.3% vs. 9.4% vs. 7.8%) (19, 21, 35). Interestingly, a generally lower frequency of flare in inflammatory arthritis compared to systemic RDs (like SLE and Behcet’s disease) was observed in the study of Fan et al. In their research, the flare rates of RA and psoriatic arthritis were 9.4% and 3.9%, respectively. In comparison, the flare rate of SLE and Behcet’s disease were 10.6% and 11.5%, respectively (19). However, this condition didn’t match the results of some other studies. In a study that involved 126 RD patients, only three patients experienced disease flare after vaccination, all of whom were diagnosed with inflammatory arthritis (27). In another study, the flare rate of inflammatory arthritis was comparable with that of systemic RDs, including SLE, SSc, Sjogren’s syndrome, and myositis (21).

### Vaccine Schedule

For RD patients, protocolized two-dose vaccination schedules are needed, as the immune responses to the first dose of COVID-19 vaccine were poor due to their immunosuppressive status (38). However, in the general population, some observational studies have reported a higher prevalence of side effects after the second vaccination, especially the systemic side effects (39, 40). This condition also fits the results in RD patients (41). As for the risk of flare, most studies reported a higher risk after the second dose, while only two studies observed opposite results (16, 18, 21, 22, 24–26, 34, 35). Besides, the sample sizes of the two studies with conflicting results were both limited. Combining with that patients who have experienced flares would always refuse to continue the vaccination, these results may reveal that patients without flares after the first vaccination would also experience RD flares after the second vaccination, and this risk may be even higher. Notably, Zavala-Flores et al. revealed a higher level of RD relapse after the second dose of vaccination for patients who had experienced flares between the two doses (16). Thus, continuing the vaccination schedule may not be recommended for these patients.

## Presentation of Flare

The flare of RDs after COVID-19 vaccination predominantly presented as joint pain, stiffness, and swelling, especially for inflammatory arthritis (16, 17, 19, 21, 26, 27, 31, 32). For SLE, except for arthritis, cutaneous and mucosal manifestations, such as malar erythema, and alopecia, were also common (16, 19, 22, 26, 31). Besides, fatigue and myalgia are commonly seen in patients with flare (21, 26, 31, 32). Several studies also reported various uncommon manifestations in different types of RD patients, such as lupus pneumonitis, leukopenia, myopericarditis, Raynaud’s syndrome, and nasal ulcer (16, 21, 22).

Most flares happened quickly after the COVID19 vaccination and were presented persistently within the first week. Barbhaiya et al. reported that only 10.9% of flares occurred later than seven days after the vaccination, while 27.7% and 61.4% of flares occurred within one day and 2-7 days after vaccination, respectively (26). Visentini et al. obtained similar results, as 83% of the flare occurred within seven days (20). In the study of Zavala-Flores et al., the average time interval between vaccination and flare was only 2.3 days (16). This time interval seems longer in the results of Connolly et al., in which the average days from the first dose of vaccination to flare rise to 6.4 (median 5, interquartile range 2-12) (21). Interestingly, this number raised to 11.4 (median 11, interquartile range, 3-20) between the second vaccination dose to flare (21).

For the severity of the flare, most patients resolved shortly after the onset (always within seven days) (16, 26, 35). Although the average resolve time in the study of Connolly et al. was up to 13 days, some patients with mild flares may not be included and extended the time to resolve, based on the fact that only flares requiring treatment were defined as involved in this study (21). On the other hand, some studies have noted that only a small part of the RD patients with flares after COVID-19 vaccination need treatment adjustment. Fan et al. found that the flare rate of disease after inactivated COVID-19 vaccination was 10%. However, less than 4% of patients required treatment escalation, much fewer comparing to the number of flare rates (19). Sattui et al. also observed a similar tendency, as flares of RDs were reported by 13.4% of patients, with only 4.6% requiring a new or increased dose of medication to treat the flare (28). In addition, the proportions of patients requiring hospitalization were even less. In the study of Zavala-Flores et al., only 10% of the SLE patients with flares needed hospitalization (16). Another study reported 151 patients with flares after vaccination, in which only 35 patients required treatment escalation, and the percentage of hospitalization even dropped to zero (21). Based on these, most of the flares after COVID-19 vaccination were mild or moderate; only a small part of the RD patients with the flare was severe. The results of Barbhaiya et al. also supported this. It founded that only 15.4% of the patients with flare reported severe symptoms after the first dose of COVID-19 vaccination, and this percentage decreased to 10.6% after the second dose (26). Besides, some studies analyzed disease activity before and after vaccination and found no significant change in the overall disease activity of RDs, thus indirectly providing evidence of non-significant flare-up (23, 34, 41–47).

## Risk Factors and Protective Factors for Flare

### Disease Activity

In 2019, the European League Against Rheumatism (EULAR) recommended that vaccination in patients with RDs should be promoted during a quiescent state of disease to avoid flare-ups and favor a good immune response (48). For COVID-19 vaccination, Fan et al. also demonstrated that the disease under reasonable control was the protective factor for self-reported disease flare only (19). However, many RD patients with moderate-high disease activity also received the vaccination due to the severe pandemic, and an increased risk of side effects after vaccination was observed in these patients (18). Besides, RD patients with flare history within one year were consistently associated with an increased risk of flare after COVID-19 vaccination (16, 21, 25, 31). Thus, it is recommended that patients with RD are having inactive disease or low disease activity before vaccination to reduce the risk of flare (3, 48, 49).

### Treatment

RD patients always need therapeutic regimens, like corticosteroids, conventional disease-modifying antirheumatic drugs (cDMARDs), and biologics, to keep the disease under control. Connolly et al. reported that patients received cDMARDs (incidence rate ratio (IRR) 0.52; 95%Cl 0.34-0.8) and biologics (IRR 0.6; 95%Cl 0.39-0.93) had lower incidences of flare (21). However, the combination of cDMARDs and biologics was associated with a higher risk of flare (IRR 1.95; 95%Cl 1.41-2.68) (21). The combination therapy may suggest the higher disease activity. However, whether it is an independent risk factor for flare is still unclear. In another study, biologics didn’t show any protection against flare, as the frequency of flare within one month after vaccination shows no difference between the biological and non-biological group (22). As for the corticosteroid, bivariate analysis in the study of Pinte et al. revealed a positive association between taking corticosteroids and disease flare after vaccination (25). A similar tendency was also observed in Connolly’s study (21). Whether this means a higher disease activity or just a predictor of flare still needs to be determined. Notably, some guidelines recommended tapering the treatment for a short period before and after each vaccination dose to enhance immunogenicity (3, 49, 50). However, treatment discontinuation may increase the flare risk of RDs. Fragoulis et al. reported a marginal association between treatment discontinuation due to COVID19 vaccination with disease flare (24). Pinte et al. also reported a higher risk of flare in patients who stopped their treatment; even the difference didn’t reach a statistical significance (4/31 vs. 21/385, *p*=0.105) (25).

Moreover, a recent clinical trial observed more flares of RA (clinical disease activity index [CDAI] criteria >10) in the group of patients who discontinued methotrexate treatment (51). On the contrary, several studies also reported a similar flare frequency among the two groups (30, 32), and the overall change in disease activity didn’t show any significant difference (42). Based on these, weighing up the risk of disease flare is still needed before stopping the medication, even if there is no solid evidence that holding therapies would cause a higher risk of disease flare.

### Other Factors

Rider et al. reported higher risks of flares for SLE, psoriatic arthritis, and polymyalgia rheumatica compared to RA (29), while some other studies found that the type of RDs had no significant effect on the occurrence of flares after vaccination (19, 21). However, another study also revealed an association between the flare-ups and having more than one RDs (25). As more than one RDs may associate with either higher disease activity or more complexity in immunity, the exact effect on disease flare is still unknown. Several other risk factors, like elderly, female, allergic history, previous infection of SARS-CoV-2, and serious reaction to a non-COVID-19 vaccine, were also reported (19, 21, 29), even though the evidence was weak.

## Association Between Flare and Vaccination

Although some patients reported flare after vaccination, the comparison between patients with vaccination and those without vaccination didn’t reveal any significant association between vaccines against SARS-CoV-2 and flare. Li et al. conducted a study of 5493 RA patients, showing no significant association between arthritis flare and the complete vaccination of mRNA (adjusted IRR 0.86, 95%CI 0.73-1.01) or inactivated virus (adjusted IRR 0.87, 95%CI 0.74-1.02) COVID-19 vaccines (44). Connolly et al. also found that mRNA vaccine was not associated with flare (IRR 0.98, 95%Cl 0.72-1.32; *p*=0.9) (21). In Pinte’s study, the incidence of RDs flare in the vaccinated and non-vaccinated groups was comparable (6% versus 8%, *p*=0.302) (25). Besides, the two groups showed no significant difference even in the length of flare-up and the incidence-densities of flare-up (25).

## The Flare Risk After SARS-CoV-2 Infection Compared to the Flare Risk After the COVID-19 Vaccination

COVID-19 vaccine and SARS-CoV-2 infection partly shared mechanisms for triggering RD relapse (7, 8). As shown above, the vaccine showed no direct association with flare, while SARS-CoV-2 infection was reported as an independent risk factor for RD flare in some studies (52–54). The flare rate of RD after SARS-CoV-2 infection presented a vastly higher flare risk than that after vaccination (52–58), with most flare rates being higher than 20% and some even higher than 40%. This may also encourage RD patients to receive the COVID-19 vaccination. It should be noted that only a limited number of RD patients got SARS-CoV-2 infection, and more studies are needed to reveal the actual situation.

## Perspectives

In summary, current evidence does not support increased risk of disease flare in RD patients after COVID-19 vaccination. However, the disease flares of RDs may be triggered by COVID-19 vaccination, especially in patients with high disease activity. Most of these flares after vaccination are mild and need no treatment escalation. Considering the benefits and risks, RD patients should receive the COVID-19 vaccination but should be vaccinated when the RDs are in stable states.

However, it is advised that there are several critical questions remain to be answered in these patients. First, which type of vaccine should be used? Based on current evidence, there is no priority for each of the three types of vaccines from the perspective of disease flare risk. Most of the studies were mainly including patients receiving the mRNA vaccine, and the data about the inactivated and adenovirus vaccines are limited. Thus, more data on the flares after vaccination with the other types of vaccine are needed. Second, is it necessary to hold the RD medication for a short time? As the relationship between treatment discontinuation and flare is still ambiguous, it’s hard to weigh up the risk of flare-up and defect immunogenicity. Future in-depth studies should focus more on these questions to help make better choices for these patients.

## Author Contributions

YX and YaL conducted the literature search, study selection and manuscript writing. YiL proposed the concept and revised the manuscript. All authors contributed to the article and approved the submitted version.

## Funding

The study was funded by the post-doctoral research funding of West China Hospital, Sichuan University (Grant number 19HXBH036), and the Science & Technology Department of Sichuan Province funding project (No.2020YJ0022). 

## Conflict of Interest

The authors declare that the research was conducted in the absence of any commercial or financial relationships that could be construed as a potential conflict of interest.

## Publisher’s Note

All claims expressed in this article are solely those of the authors and do not necessarily represent those of their affiliated organizations, or those of the publisher, the editors and the reviewers. Any product that may be evaluated in this article, or claim that may be made by its manufacturer, is not guaranteed or endorsed by the publisher.
