# Supplementary figures and images for: The Flare of Rheumatic Disease After SARS-CoV-2 Vaccination: A Review
Source: Front Immunol. 2022 Jul 4;13:919979. doi: 10.3389/fimmu.2022.919979 (PMC9289284; doi:10.3389/fimmu.2022.919979)

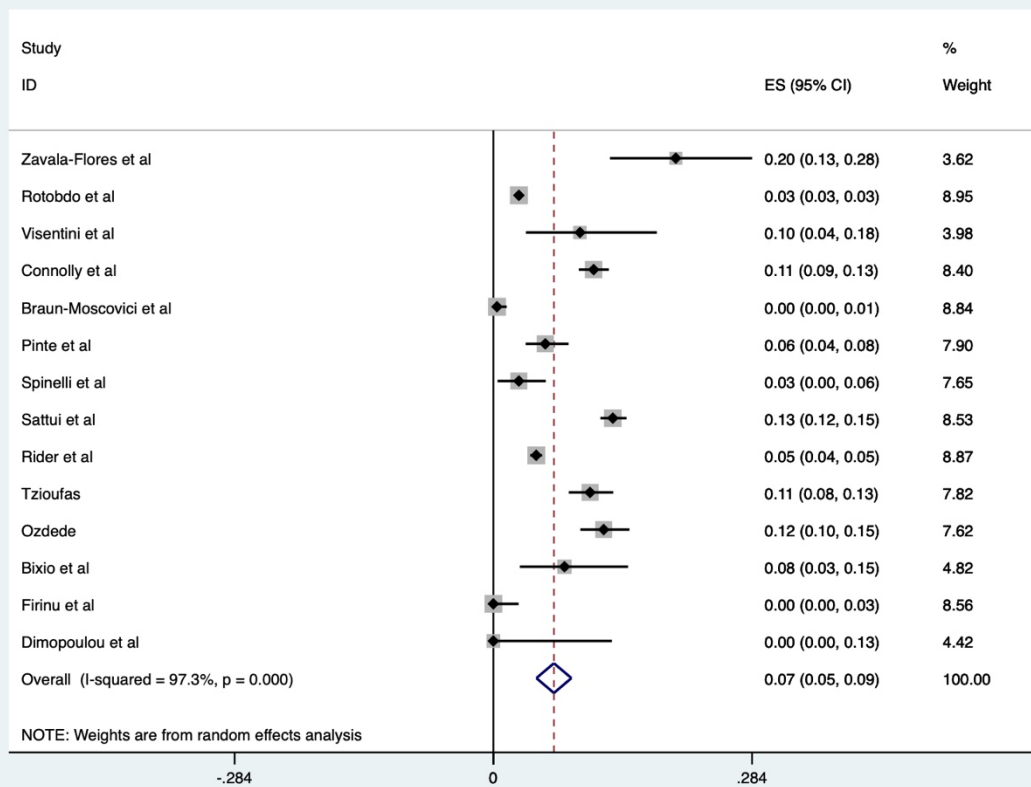

**Supplementary Figure 1.** Flare rate of rheumatic disease after mRNA COVID-19 vaccination.

Supplement: Supplementary file 1 [file Image_1.pdf]
