# Supplementary material for: The Flare of Rheumatic Disease After SARS-CoV-2 Vaccination: A Review
Source: Front Immunol. 2022 Jul 4;13:919979. doi: 10.3389/fimmu.2022.919979 (PMC9289284; doi:10.3389/fimmu.2022.919979)
Supplement: Supplementary file 2 [file Image_2.pdf]

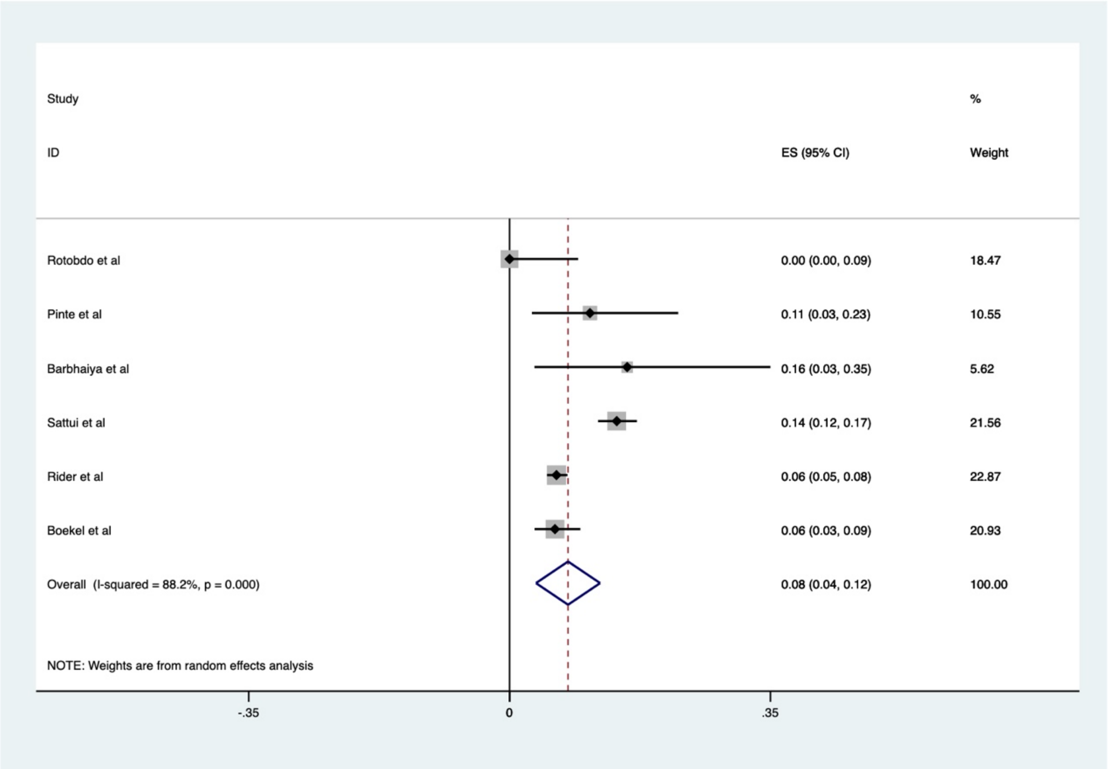

Supplementary Figure 2. Flare rate of rheumatic disease after adeno-virus based COVID-19 vaccination.
